# The role of radiotherapy in multimodal treatment of pancreatic carcinoma

**DOI:** 10.1186/1748-717X-5-64

**Published:** 2010-07-08

**Authors:** Thomas B Brunner, Martin Scott-Brown

**Affiliations:** 1 Gray Institute for Radiation Oncology & Biology, University of Oxford, Oxford, UK

## Abstract

Pancreatic ductal carcinoma is one of the most lethal malignancies, but in recent years a number of positive developments have occurred in the management of pancreatic carcinoma. This article aims to give an overview of the current knowledge regarding the role of radiotherapy in the treatment of pancreatic ductal adenocarcinoma (PDAC). The results of meta-analyses, phase III-studies, and phase II-studies using chemoradiotherapy and chemotherapy for resectable and non-resectable PDAC were reviewed. The use of radiotherapy is discussed in the neoadjuvant and adjuvant settings as well as in the locally advanced situation. Whenever possible, radiotherapy should be performed as simultaneous chemoradiotherapy. Patients with PDAC should be offered entry into clinical trials to identify optimal treatment results.

## Introduction

Despite considerable progress in oncology, the poor prognosis of patients with pancreatic ductal adenocarcinoma (PDAC) has not significantly improved. In Europe and the USA, PDAC is still ranked fourth and in the UK sixth for cancer associated mortality[1,2]. More than 80% of patients with PDAC present with irresectable disease. One third of these patients have locally advanced pancreatic carcinoma (LAPC), the rest have distant metastases. In this article we review the role of radiotherapy (RT), currently used as chemoradiotherapy (CRT) in the management of pancreatic cancer.

We discuss its neoadjuvant use in improving resectability rates, its adjuvant use in maintaining local control and its role as primary treatment of LAPC. To identify eligible studies we undertook a Medline database search from 1980 to 2008 and also included unpublished meeting reports of phase III trials. Wherever possible we focused on the data available from prospectively randomised phase III trials. However for some aspects of treatment, data was not available from prospectively randomised phase III trials and therefore some studies with a lower degree of evidence needed to be included. Before addressing the respective treatment situations (adjuvant, neoadjuvant or definitive) general matters concerning all stages of disease are highlighted in the introduction.

### Diagnostic imaging and disease staging

A number of imaging modalities may be useful in arriving at a diagnosis in patients presenting with symptoms or signs suggestive of pancreatic carcinoma[3]: Ultrasound, Endoscopic UltraSound (EUS), Endoscopic Retrograde CholangioPancreatography (ERCP), multidetector computed tomography (CT) and Magnetic Resonance Imaging (MRI) including Magnetic Resonance CholangioPancreatography (MRCP). The two most sensitive techniques to detect pancreatic carcinoma are multidetector CT and MRI in combination with MRCP. If performed by experienced investigators EUS can reach even higher sensitivity. It is recommended that centres use the imaging modality with which they have most experience. Biopsy proof of a pancreatic mass is not required if the mass is resectable except within neoadjuvant treatment protocols. However, histological proof is mandatory prior to the initiation of any palliative treatment. Tissue can be obtained either via EUS or CT-guided biopsy or percutaneously[4]. For preoperative assessment of local tumour spread and resectability, multi-detector CT and EUS represent the best staging tools[5]. Additionally, a chest x-ray is required if the thorax is not included in the CT scan of the abdomen.

### Surgical technique and histopathological analysis

Resectability is the most important initial therapeutic decision. The infiltration of adjacent organs, e.g. duodenum or stomach, in itself does not exclude resection. Very often resectability depends on vascular involvement [6]. Tumours infiltrating the coeliac trunk or the superior mesenteric artery are very rarely resectable whereas infiltration of the portal vein often does not exclude resection. However a systematic review has shown that even if the tumour is technically resectable the 5 year survival rate of patients undergoing resection with involvement of the portal and superior mesenteric veins is very low[7]. Infiltration of the superior mesenteric vein often prevents a margin negative (R0) resection. Preoperative biliary drainage with a stent is only necessary when the patient suffers from cholangitis or if the operation cannot be performed immediately. The recommendations to surgeons so as to achieve an R0 resection is to allow an excision margin of 10 mm for pancreatic tissue, bile ducts, and stomach [8], however for anatomic reasons there are no definite recommendations for the retroperitoneal margin. For pancreatic head tumours the resection consists of a duodenopancreatectomy with or without preservation of the pylorus. In rare cases when the carcinoma extends to the body of the pancreas a total pancreatectomy may be necessary. Classic Whipple's procedure and pylorus preserving techniques are equivalent with respect to post-operative complications and lethality as well as long-term survival [9]. Distal pancreatectomy is performed for tumours of the pancreatic tail, whilst tumours of the pancreatic body require a subtotal distal pancreatic resection or a total duodenopancreatectomy. Radical extended lymphadenectomy has not been proven to result in prolonged survival [10]. Standard lymphadenectomy for a Whipple's procedure for a tumour of the pancreatic head comprises the following compartments: Complete and circular resection of the nodes of the hepatoduodenal ligament, around the common hepatic artery, around the portal vein and the cranial aspect of the superior mesenteric vein. Furthermore, the right coeliac trunk lymph nodes and the right hemi-circumference of the trunk of the superior mesenteric artery are dissected. Resection should be abandoned if distant metastases are encountered intraoperatively.

The pancreatic margin and the margin of the biliary duct should always be histologically evaluated intraoperatively (e.g. by frozen section). Putative liver metastases or peritoneal carcinomatosis should also be evaluated intraoperatively with frozen section. The full histological report should include: pT, pN, number of analysed lymph nodes, nodal micrometastases, R-classification (tumour status at the resection surface to the remaining pancreas, retroperitoneal tumour status) and lymphovascular invasion, vascular invasion and perineural invasion [8].

Histological assessment to diagnose a clear resection margin (R0) should include the hepatic duct and the pancreatic resection surface (including the retroperitoneal margin). To facilitate orientation the retroperitoneal margin should be ink-marked at the time of resection.

### Radiotherapy technique, quality assurance, target volume selection, dose fractionation and normal tissue toxicity

Radiotherapy for pancreatic carcinoma should always be performed as chemoradiotherapy, except for the rare cases of palliative treatment of bone metastasis. Palliative analgesic treatment of the pancreas with hypofractionated radiotherapy only (20 Gy in 5 fractions) is used in the UK however there is no evidence from the literature supporting this practice. Patients who are not treated within clinical trials should have infusional 5-fluorouracil (5-FU) or capecitabine for chemoradiotherapy [8]. In order to avoid toxicity, radiotherapy should be conventionally fractionated (1.8 - 2 Gy/fraction, total dose 50 to 55 Gy). However a hypofractioned regimen (30 Gy in 10 fractions) was reported to be safe by the MD Anderson Cancer Center group[11]. Treatment planning should be 3D-conformal and IMRT is recommended. Total dose was increased up to >60 Gy using multiple field techniques or IMRT together with restrictive target volume definitions[12] however we recommend to keep total doses to the primary tumour to 50 - 55 Gy in conventional fractionation outside of clinical studies. The most important normal tissue toxicities encountered or to be avoided are haematotoxicity, duodenal and gastric ulceration/bleeding, diarrhoea, renal and hepatic toxicity. Recommended dose limits for critical organs are:

• Liver: 50% of the volume ≤30 Gy

• Kidneys: One kidney no more than maximally 50% of the volume should receive >20 Gy Other kidney no more than maximally 30% of the volume should receive >20 Gy

• Spinal cord: ≤45 Gy.

To date there is no consensus as to whether regional lymphatics need to be emcompassed in the irradiation target volume and if so which areas need to be covered. Up to 80% of all resectable pancreatic head tumours have regional lymphatic metastasis, which suggests that inclusion of lymphatics may enhance locoregional control. The distribution of the frequency of metastases in 175 resectable tumours was posterior pancreaticoduodenal area (37%), superior (25%) and inferior margin of the pancreatic head (24%), anterior pancreaticoduodenal area (23%), upper para-aortic (22%), hepatoduodenal ligament (18%), superior margin of the pancreatic body (11%), and superior mesenteric artery(10%)[13]. Most of these regions, with the exception of the hepatoduodenal ligament and the upper paraaortic nodes, are probably within the 80% isodose volume even when elective nodal irradiation is not being carried out, as hypothesised by Murphy et al[14]. The field design in pancreatic carcinoma requires diligence and the total volume needs to be carefully restricted to avoid unnecessary toxicities. With a careful field design the primary tumour and the elective lymphatics can be encompassed in a treatment volume below 600 mL in most patients [13], e.g. in our institution the maximum planning target volume (PTV) that we allow is 800 mL but most patients will have a smaller volume. The tolerance of gemcitabine based concurrent CRT is highly dependent upon the total treatment volume in pancreatic carcinoma [15]. Excess toxicities have been reported if the radiotherapy techniques have not paid sufficient attention to these rules. Careful, active supportive therapy is essential in ensuring the tolerability and effectiveness of concurrent CRT. This comprises stenting of the common bile duct, anti-emetic treatment, proton-pump inhibitor therapy, analgesia and parenteral feeding if necessary. When radiotherapy techniques are unfamiliar or complex, as in pancreatic carcinoma, problems with the quality of the delivered radiotherapy are more likely. Quality assurance in radiotherapy for pancreatic cancer should comprise standard dosimetry assessments, adequate PTV coverage and adequate protection of normal tissue structures [16]. An example to highlight the significance of radiotherapy quality assessment is the lack of a description in the protocol for radiotherapy technique, normal tissue constraints and quality assessment within the adjuvant ESPAC-1 trial where chemoradiotherapy appeared to be detrimental to the patient[17]. This detrimental effect could be due to both geographical miss and increased radiotherapy toxicity.

## Adjuvant Chemoradiotherapy

Adjuvant (after R0-resection) or additive chemoradiotherapy (after R1- or doubtful complete resection) aims to prolong survival by improved local tumour control. A total of seven randomised controlled trials have been published to date. The most important of these are the Gastrointestinal Tumour Study Group (GITSG) trial[18], the European Study Group for Pancreatic Cancer (ESPAC-1) trial[17], the Charité Onkologie (CONKO-001) trial[19] and recently the Radiation Therapy Oncology Group (RTOG 97-04) trial[20]. We have not included the small Norwegian Pancreatic Cancer Trial Group study [21] and the EORTC trial where about half of the patients had periampullary and not pancreatic carcinoma [22]. The trial that is likely to have had the largest impact on adjuvant chemoradiotherapy in UK practice is the ESPAC-1 trial of adjuvant treatment for pancreatic cancer[17,23]. This trial recruited patients from 53 hospitals in a 2 × 2 factorial design. There were four study groups which included (1) surgery only (n = 69), (2) chemotherapy with 5-FU (5-FU bolus and leucovorin d1-5q29 for six cycles) (n = 73), (3) 5-FU based chemoradiotherapy according to the GITSG [18] method (n = 73), and (4) both treatments (chemoradiotherapy followed by chemotherapy) (n = 73). The randomisation process of ESPAC-1 was rather complicated. In the first report of this trial[23] non-randomised patients were included, who had been allowed to choose their treatment option, and we will therefore not discuss the results further. However in the second report only correctly randomised patients were included into the analysis[17]. The survival results were given as five-year survival rates which were 8% for patients not receiving chemotherapy, 21% for those receiving chemotherapy, and 10% among patients who received chemoradiotherapy. However the interpretation of the data is very difficult because the control groups contain a mix of subpopulations, e.g. chemotherapy is compared to patients having had surgery only or having had chemoradiotherapy. The authors concluded that adjuvant chemotherapy prolonged survival whereas chemoradiotherapy had a negative effect on survival. Patients with adjuvant chemoradiotherapy alone achieved a median OS of 13.9 months, lower than the reported 16.9 months after surgery alone. In ESPAC-1, survival after adjuvant chemoradiotherapy was shorter than the median OS (15 months) of patients with LAPC who were treated with combined chemotherapy and chemoradiotherapy without resection [24]. The report says that chemoradiotherapy was performed according to 'local quality-assurance procedures in place', and this might explain the detrimental effect of chemoradiotherapy. The radiotherapy delivered in this trial was suboptimal using a technique which was conceived in the early 1970s: the split-course technique, prolonging treatment time, is known to reduce the rate of local control. The total dose employed was only 40 Gy, and 5-FU was given as a bolus injection schedule which is known to be inferior to prolonged intravenous infusional schedules. 3-D conformal treatment planning techniques were available at the time when the study was conducted but were not used, and even simple parallel opposed Ant.-Post. techniques were allowed within this trial. A good example of how improvements in quality assurance and technique can affect chemoradiotherapy in upper gastrointestinal tumours is the successful introduction of such techniques into the adjuvant treatment for stomach cancer [25]. Therefore we believe that the ESPAC-1 trial cannot answer the question as to the role adjuvant chemoradiotherapy plays in the treatment of resected pancreatic carcinoma.

The results of the RTOG 97-04 study were published in 2008 [20]. This study tested whether the effect of adjuvant 5-FU based chemoradiotherapy (50.4 Gy; 250 mg 5-FU/m²/day continuous infusion) could be enhanced by adding gemcitabine for three weeks pre-CRT and for 12 weeks post-CRT (G-arm) whereas the control arm contained pre-CRT and post-CRT 5-FU chemotherapy. The principal question was therefore chemotherapeutic and randomisation included stratification according to tumour diameter (<3 cm vs ≥3 cm), nodal status, and surgical margins.. In contrast to the ESPAC-1 study, prospective quality assurance of radiotherapy was required for all patients and current radiotherapy techniques were used. The analysis comprised 442 patients. The median overall survival (OS) of patients with tumours of the pancreatic head was significantly longer in the G-arm compared to the control arm with 5-FU chemotherapy (20.6 vs. 16.9 months, 32% vs. 21% after 3 years; p = 0.033) however not if tumours of the pancreatic body or tail were included. On multivariate analysis three parameters reached statistical significance: the treatment arm (p = 0.025), the nodal status (p = 0.003) and the maximal tumour diameter (p = 0.03). The non-haematological toxicity (>Grade 3) did not differ between the two arms. Grade 4 haematotoxicity was 14% in the gemcitabine arm and 2% in the 5-FU arm, without any difference in the rate of febrile neutropenia. This trial, has changed standard adjuvant therapy in the US for tumours of the pancreatic head: Currently, for the National Cancer Institute, standard treatment is radical pancreatic resection with or without post-operative 5-fluorouracil chemotherapy and radiation therapy[26]. For the National Comprehensive Cancer Centers Network (NCCN), standard adjuvant treatment options are systemic gemcitabine followed by chemoradiation (5-FU-based) *or *chemotherapy alone (gemcitabine preferred or 5-FU/leucovorin or capecitabine)[27].

Compared to ESPAC-1, RTOG 97-04 included patients with a more unfavourable distribution of risk factors (resection status, pN-category and largest tumour diameter) but nevertheless resulted in longer survival. Superior quality and technique of chemoradiotherapy may explain this difference. The improved radiotherapy technique employed in the RTOG trial is reflected in the reduction of local recurrence rates being 25% in the RTOG trial compared to 47% in the GITSG trial and 62% overall in the ESPAC-1 trial. Remarkably, the rate of positive margins after resection was almost twice as high in the RTOG-trial (35%) compared to the CONKO-001 trial (19%) in the respective experimental arms but this was without a reduction of survival in this comparison, probably due to the radiotherapy element. The CONKO-001 trial comparing chemotherapy only with observation reported a local recurrence rate of approximately 38% in both arms (34% with adjuvant treatment and 41% without) which is considerably higher compared to the RTOG trial. Of course, many of these local recurrences are not isolated. Therefore it can be hypothesised that more effective systemic treatment controlling systemic disease could benefit considerably from combination with effective radiotherapy. Despite a median disease-free survival time of 13 months with adjuvant gemcitabine vs 7 months without (p < 0.001), there was not a statistically significant difference in overall survival in the CONKO-001 trial. The disease-free survival effect was observed in both the R0 and the R1 resection subgroups.

To date, there are two meta-analyses addressing adjuvant chemoradiotherapy in pancreatic carcinoma which result in discrepant conclusions. The discrepancies could be explained by the predominance of the randomised and non-randomised ESPAC-1 patients in the meta-analysis from Stocken et al [28] on the one hand and by the inclusion of the non-randomised Yeo et al data [29] in the meta-analysis published by Khanna et al [30]. Furthermore, relative weight was taken into account in the Khanna report but not in the Stocken report. Thus each of the meta-analyses has flaws in their design. The recent meta-analysis by Khanna et al [30] investigated the effect of adjuvant chemoradiotherapy compared with surgery only. Five prospective studies with a total of 607 patients (229 resected vs. 378 resected plus chemoradiotherapy) were included. The 2-year OS rates reached 15-37% after resection alone and 37-43% after resection and adjuvant chemoradiotherapy. The percentage gain in survival from adjuvant chemoradiotherapy was 3% - 27% despite the absence of a statistically significant prolongation of survival in any of the individual studies. In total, an absolute gain in survival of 12% was calculated after 2 years (95% CI, 3%-21%, p = 0.011). However, the relative prolongation of survival decreased with more modern studies over time and did not reach statistical significance in the latest trials. A second meta-analysis from Stocken et al [28] analysed adjuvant chemoradiotherapy and adjuvant chemotherapy. Adjuvant chemotherapy improved survival in patients with R0 resections but this benefit was not seen with adjuvant chemoradiotherapy. The group concluded that adjuvant/additive chemoradiotherapy is only more effective than chemotherapy after R1-resections. For this patient group the meta-analysis showed a reduction of the hazard ratio by 28% (s.d. 19), whereas adjuvant chemotherapy showed no significant effect on survival. A retrospective study in additive chemoradiotherapy by Wilkowski et al after R1-resections also reported excellent survival data [31].

In summary, the value of adjuvant therapy is currently a matter of great controversy. To date, seven randomised phase III studies on the use of adjuvant CRT and adjuvant chemotherapy have been conducted, the most important of which are summarised in Table 1[17-19,22,23]. The fully published studies which comprise CRT [17,18,22,23] have substantial shortcomings as to the design and the realisation of radiotherapy as discussed in detail elsewhere [32]. Therefore, the efficacy of adjuvant CRT according to current quality standards is uncertain. This unsatisfactory situation on the significance of adjuvant CRT can be summarized in the following way:

**Table 1:** Phase III-studies for adjuvant therapy

Group - Study Year	Patients (n)	Inclusion criteria Resection-Status	Treatment arms	Median overall survival (Months)	*p*-value	Preoperative imaging
GITSG-1985[18]	49	R0	CRT Observation	21.0 10.9	0.005	No

EORTC-1999[22]	114*	R0	CRT Observation	17.1 12.6	0.099	No

ESPAC-1-2004[17]	289^#^	R0 or R1	Cx No Cx^&^	21.6 16.9	Not available	No

CONKO-001-2007[19]	368	R0 or R1	Cx Observation	22.1 20.2	0.06	Yes

RTOG 9704 2008[20]	442^	R0 or R1	CRT + GEM CRT + 5-FU	20.6 16.9	0.033	Yes

Abbreviations: 5-FU = 5-fluorouracil, CRT= chemoradiotherapy, Cx= chemotherapy, GEM= gemcitabine, n= number, R0 = clear resection, R1 = microscopically positive margins. Median overall survival rates from five randomized studies in patients with resected pancreatic carcinoma. None of these studies employed postoperative imaging to exclude tumour persistence or distant metastasis. *The EORTC study included 218 patients with periampullary and pancreatic carcinoma. The figures in the table are based upon the 114 patients with pancreatic carcinoma. ^#^The ESPAC-1 study included 541 patients, but only 289 were included into the 2 × 2 factorial randomization. Arms: observation, chemotherapy, chemoradiotherapy, chemoradiotherapy followed by chemotherapy. The survival rates are given for the best treatment arm (chemotherapy) and observation. ^&^The comparison arm comprises both, patients with observation and patients with chemoradiotherapy. ^The RTOG 9704-study included a total of 442 patients, 380 of them had pancreatic head tumours.

• The European and the American trials are based upon different treatment paradigms. Whilst in North America CRT is regarded as one of the options of standard of care based on the GITSG- and RTOG-data [18], in Europe adjuvant chemotherapy is preferentially given.

• To define the role of adjuvant CRT, a large randomised study exploiting the full options of the respective therapeutic modules needs to be planned carefully and interdisciplinary. Such a study should be accompanied by intensive therapeutic monitoring and be stratified by tumour location, resection status, nodal status and tumour size as identified in the RTOG 97-04 trial.

### Summary

After R0-resection, patients should receive adjuvant chemotherapy in the light of the relatively weak data to support the use of adjuvant CRT. Therefore patients should not be treated with CRT outside of clinical trials in the adjuvant situation. Those patients most likely to benefit from adjuvant CRT would be expected to be patients with tumours of the pancreatic head, pN1-status and a maximal tumour diameter of >3 cm. Additive CRT should be considered after R1-resection.

## Neoadjuvant therapy

The concept that neoadjuvant CRT may be more effective than adjuvant CRT has been supported recently in resectable rectal carcinoma with a high risk for local relapse (German Rectal Cancer Group). Neoadjuvant chemoradiotherapy resulted in tumour regression causing a higher rate of R0-resections, improved local control and lower long term toxicity compared with post-operative chemoradiotherapy [33]. In pancreatic carcinoma the situation may be similar: general radiobiological considerations suggest increased efficacy of preoperative treatment due to a more effective chemotherapy delivery with an intact blood supply, compared to the reduction of blood flow and increased hypoxia in the post-operative situation. Hypoxia is one of the most important factors of radiation resistance [34]. Another problem in post-operative treatment is that the gastrointestinal reconstruction receives the full dose of radiotherapy and postoperatively dose is therefore limited to avoid injury to the anastomosis of the reconstructed bowel. Beyond these radiobiological considerations there are clinical implications supporting the hypothesis that neoadjuvant chemoradiotherapy could be more effective than adjuvant treatment[35]: (1) Timely access to adjuvant therapy is problematic after pancreaticoduodenectomies because delayed post-operative recovery often does not allow patients to start within 6 to 8 weeks of surgery [20]. (2) Neoadjuvant therapy may allow better selection of patients appropriate for surgical resection. Patients with aggressive tumour biology and early evidence of metastatic disease during the time of neoadjuvant treatment can be spared a surgical procedure. This can be illustrated with the overall survival curves of two adjuvant phase 3 studies: the CONKO-001 and the ESPAC-1 trial, both show a complete overlap of the survival curves during the first 12 months after surgery pointing to ineffective therapy for patients with an aggressive tumour biology[17,19]. The same observation can be made in a preoperative ECOG phase II trial including 53 patients which reported six patients (11%) with distant metastasis precluding surgery after preoperative chemoradiotherapy [36]. (3) Neoadjuvant therapy may improve R0 resection rates due to killing of cancer cells beyond the macroscopically visible tumour and so decrease local failure rates as suggested by intra-institutional comparisons between pretreated and non-pretreated patients at the MD Anderson Cancer Center and the Fox Chase Cancer Center [11,37]. Neoadjuvant therapy may also reduce the number of positive nodes [38].

The efficacy of neoadjuvant treatment can only be deduced from phase II-studies or retrospective reports because no prospectively randomised phase III-study has been published at present. In a prospective comparative study at the Mount Sinai Hospital in New York City [39] laparotomy and/or CT followed by EUS, angiography or laparoscopy was used to determine potential resectability prior to therapeutic intervention. Patients with locally invasive tumours deemed to be non-resectable as defined in the report (T3, N0-1, M0; TNM 1997; n = 68) were treated with split-course-chemoradiotherapy (5-FU, streptozotocin and cisplatin) and subsequently surgery if rendered amenable to resection. It should be noted that conventionally non-resectable tumours are defined as stage III (T4 N0-1 M0). Resectable tumours (T1-2, N0-1, M0; n = 91) underwent immediate pancreaticoduodenectomy. Sixty-three of 91 patients received adjuvant radiotherapy or chemotherapy. Thirty of 68 patients with initially irresectable tumours underwent surgery with downstaging observed in 20 patients. The first CT re-staging was performed after 10 weeks. Delayed response on CT scans after chemoradiotherapy has often been reported and repeated reassessment of resectability could have increased resectability rates in this trial. The median OS time of all patients receiving preoperative treatment was 23.6 months compared to 14.0 months for patients who had initial tumour resection (p = 0.006). This is an unexpected result and possible explanations could be that chemotherapy was continued routinely after chemoradiotherapy resulting in a median OS of 18 months in the patients without surgery. The chemoradiotherapy regimen was unusual with 54 Gy and two splits as well as combined 5-fluorouracil, streptozotocin, and cisplatin and it is not clear how much this has contributed to the effectiveness in the experimental arm. On the other hand the 14 month median OS for the primary resected patients is considerably lower compared to the observational arm of the recently published CONKO-001 trial. Part of this may be explained by surgical technique.

A group of 86 patients were treated with neoadjuvant chemoradiotherapy (30 Gy in 10 fractions) with concurrent gemcitabine (400 mg/m²/week) at the M.D. Anderson Cancer Center [11]. Surgery was performed eleven to twelve weeks later. This resulted in 74% of the patients undergoing tumour resection. Better efficacy was observed after gemcitabine concurrent with radiotherapy [11] compared to previous studies from the same group combining radiotherapy with 5-FU [40,41] Pathological tumour response grading was ≥50% in 58% of the resected tumours. Median OS time of the patients was 36 months. At the Duke University Medical Center in Durham, North Carolina, 111 patients with non-metastatic pancreatic carcinoma were treated with chemoradiotherapy (45 Gy, 5.4 Gy Boost, 5-FU/MMC/cDDP) [42]. Seventy-two percent had a R0-resection and 70% were staged ypN0 (y = posttreatment). The total survival rate of the resected patients was 32% after 2 years. In our own experience 58 patients were resected without any neoadjuvant therapy and had a median OS of 21 months whereas 21 patients with initially unresectable tumours underwent CRT followed by resection and had a median OS of 54 months [43]. The small number of patients in the group with neoadjuvant chemoradiotherapy is of course a limiting factor in this comparison as reflected in the p value (p = 0.084). A summary of the relevant studies to date is shown in Table 2. Very recently, a systematic review and meta-analysis on neoadjuvant therapy in 4,394 patients (CRT in 94% and chemotherapy in 6%) showed that those patients categorised to be non-resectable before treatment but having resection after neoadjuvant treatment had comparable survival (median overall survival 20.5 months) to patients with initially resectable tumours (median overall survival 23.3 months) [44].

**Table 2:** Selected studies of neoadjuvant chemoradiotherapy

Study Institution	Number of patients	Total dose of RT (Gy)	Chemo-therapy	Median OS (months)	1(2,4,5)-year-OS-rate (%)	Rate of local recurrence (%)	Rate of resectability (%)	Rate of clear resections
Hoffman et al. 1998[36,74] multi-centric*	53	50.4	5-FU/MMC	9.7 all pts 15.7 res	*2y: *27 res	24/53 (26%) 3/24 (13%) res	24/53 (45%)	n.a.

Snady et al. 2000[39] Mount Sinai*	159 68* (20 res*) vs 91 adj	54 +14 Gy	5-FU/Cis/ Streptozotocin	23.6* (32 res) vs 14.0	*1 y:* 86*(89)vs 64 *2y:* 58*(60) vs 32 *3y:* 27*(40)vs 17	n.a.	20/68 (29%) -	95% neo 84% adj

Breslin et al. 2001[75] MDACC^#^	132	45 or 50.4 Gy or 10 × 3 Gy ± IORT	5-FU, or Tax or Gem	21	*1y: *75 *2y: *40 *5y: *23	8/132 (6%)	-	88%

Sasson AR et al.2003[76] FCCC*	116 61 neo 55 adj	50.4	5-FU/MMC or Gem	All 18 neo 23 adj 16	n.a.	n.a.	-	39% n.a. n.a.

White et al. 2005[68] Duke^#^	193 i.p.r.:102 i.l.a.: 91	45 +5.4 Gy	5-FU or Gem	23 39 i.p.r. 20 i.l.f.	*3y: *37 res *5y: *27 res	n.a.	70/193 (36%) 54/102 (53%) 16/91 (18%)	73% n.a. n.a.

Evans et al. pII 2008[11] * MDACC	i.p.r.: 86 (64 res)	30 Gy (in 10 fractions)	Gem (7 × 400 mg)	23 34	*1y: *92 res *2y: *62 res *5y: *36 res	7/64 (11%)	64/86 (74%)	11%

Golcher et al. 2008[43]^# ^Erlangen	79 21 neo* 58 res^	50.4 Gy +5.4 Gy	5-FU or Gem	54 neo-res 21 no CRT res	*2y: 56 neo-res* *43 no CRT res*	n.a.	21/103(20%)	90% neo 78% res

Gillen et al. meta-analysis 2010[44]	All: 4,394 i.p.r.: 32% i.l.a.: 51% both: 17%	>60%: 40-60 Gy	50% 5-FU(+) 40% Gem(+)	i.p.r.: 23 i.l.a.: 21	i.p.r/i.l.a. *1y *78/78 *2y *47/50	n.a.	i.p.r.: 74% I.l.a.: 33%	i.p.r.: 82% I.l.a.: 79%

Stessin et al. SEER 2008[35]	i.p.r.: 3,885 70 neo 1,478 adj RT 2,337 obs	n.a.	n.a.	23 neo 17 adj 12 obs	*Neo/adj/obs* *1y *79/68/50 *2y *49/34/28	n.a.	n.a.	n.a.

Abbreviations: (+) = or additional agent, 5-FU = 5-fluorouracil; adj = adjuvant therapy; Cis = cisplatin; FCCC = Fox Chase Cancer Center, Philadelphia, PA; Gem = gemcitabine; Gy = Dose in Gray; i.p.r = initially potentially resectable; i.l.a. = initially locally advanced, MDACC = M.D. Anderson Cancer Center Houston, TX; MMC = mitomycin C; n.a. = not available; neo = neoadjuvant; obs. = observation, no adjuvant therapy, OS = overall survival; res = resected patients, RT = radiotherapy; Tax = paclitaxel; vs. = versus.

*initially unresectable patients ± resection after chemoradiotherapy;

§this study indicates overall survival as explained: (1) patients with chemoradiotherapy (2) numbers in brackets: patients with chemoradiotherapy and resection (3) numbers to the right of 'vs.': patients after primary resection.

^This study compared patients with immediate tumour resection (n = 58) with non-resectable patients who subsequently underwent neoadjuvant chemoradiotherapy

The first multicenter randomised study for neoadjuvant therapy in pancreatic carcinoma is currently recruiting [45]. This study will compare the outcomes of patients treated with neoadjuvant CRT plus resection with those treated with immediate resection in individuals whose disease is considered to be resectable at diagnosis. The resection is followed by adjuvant chemotherapy in both arms. Because of the unique position of this study in the neoadjuvant setting this study is highly relevant and interested potential study centres are welcome to participate.

### Summary

No conclusions can be drawn to date regarding the use of neoadjuvant therapy. Neoadjuvant therapy is expected to prolong survival by achieving higher rates of curative resections (R0), ypN0-tumours and increased local tumour control. Patients with resectable tumours at diagnosis should not be treated with neoadjuvant CRT outside of clinical trials. In patients with locally advanced, initially unresectable tumours, chemoradiotherapy allows secondary resectability in about 10-20% of the patients.

## Locally advanced tumours

About one third of the patients with PDAC present with locally advanced pancreatic cancer (LAPC) at diagnosis. The definition of LAPC is unresectable disease in the absence of distant metastases. Recently, the NCCN Practice Guidelines in Oncology have been published and these distinguish between resectable, borderline resectable and unresectable disease [46]. Borderline resectable tumours should be regarded as LAPC because of the high likelihood of achieving an incomplete (R1 or R2) resection. Patients with LAPC are potentially curable if a clear resection (R0) can be performed after downstaging of the tumour and therefore should be treated with the intention of cure. Nevertheless, there is an ongoing controversy about optimal therapy for this group of patients.

### Chemoradiotherapy v Best Supportive Care

The advantage of chemoradiotherapy over best supportive care was tested in a small prospectively randomised trial (16 vs 15 patients)[47]. Overall survival was significantly longer, the quality of life significantly better and the rate of distant metastases significantly lower in the chemoradiotherapy group.

### Chemoradiotherapy v Radiotherapy

Early randomised studies showed that combined CRT (with total radiotherapy doses of 40 Gy and 5-FU) followed by additive chemotherapy was superior to radiotherapy alone [48,49]. In the GITSG trial [49] patients were randomised to either radiotherapy or CRT (40 Gy) or high dose CRT (60 Gy). Combined CRT was significantly superior to radiotherapy alone, with mean OS times of 10.4 vs 6.3 months. The problem with the radiotherapy techniques at that time was that large volumes of the small intestine were irradiated which lead to dose-limiting toxicity [17,50]. Even if the studies on LAPC are not fully consistent [51], there is general consensus that radiotherapy should be given concurrently with chemotherapy in LAPC.

### Chemoradiotherapy v Chemotherapy

Previous randomised phase II studies comparing CRT with chemotherapy showed some superiority of CRT in terms of local control and OS (Table 3)[50,52]. Yet, a recent report from a phase III-trial [53] resulted in inferior results following CRT. It should be noted however that the standards of radiotherapy delivery in this trial were sub-optimal with unusually high levels of radiotherapy induced side-effects. In addition the investigators used an unusual chemotherapy regimen (5-FU and cisplatin) that would not be considered standard in this setting. The results of a recently published meta-analysis [54] cannot resolve the problem of an imbalanced comparison of chemotherapy with chemoradiotherapy because this meta-analysis includes only the early trials from the 1980's and the report of the FFCD trial as discussed above [53]. The only study (ECOG4201) using modern radiotherapy techniques was reported in 2008 by Loehrer and coworkers as an abstract [55]. Thirty-eight patients treated with gemcitabine alone were compared to 36 patients treated with gemcitabine-based chemoradiotherapy. There was no difference in partial response rate in the two treatment groups, but significantly more patients achieved stable disease in the chemoradiotherapy arm (68% vs 35%). At the same time fewer patients had progressive disease in the chemoradiotherapy arm (6% vs 16%). Overall survival was statistically longer after chemoradiotherapy compared to chemotherapy (p = 0.034; 12 m-OS 50% vs 32%; 18 m-OS 29% vs 11%, 24 m-OS 12% vs 4%; mOS 11.0 vs 9.2 m). The increased toxicity of chemoradiotherapy in this study compared to chemotherapy is probably attributable to the unusually high dosing of gemcitabine (600 mg/m*/week for six weeks) in conjunction with radiotherapy. The predominant grade 3/4 toxicities were fatigue and gastrointestinal side effects. This trial was closed after recruitment of 74 patients whilst the accrual target was 316 patients.

**Table 3:** Selected studies for locally advanced pancreatic cancer employing chemoradiation ± chemotherapy

Trial (patients)	Chemotherapy*	Radiotherapy (Gray)	Median survival (months)	p-value
Johnson[59] (129)	Lithium Gamonelate	-	5.4	n.a.
Maisey[56] (44/46)	5/FU PVI 5-FU/MMC	-	32% 1 year 43% 1 year	n.a.
Louvet[60] (47/50)	Gem Gem/Ox	- -^$^	10.3 10.3	p = n.s.
Rocha Lima[61] (24/27)	Gem Gem/Iri	- -^$^	11.7 9.8	p = n.s.
Van Cutsem[62] (80/82)	Gem Gem/tipifarnib	-	8.7 11	p = n.s.
GITSG 1985[77] (25/83/86)	- ***5-FU*** ***5-FU***	60 40 60	5.2 9.6 9.2	p < 0.01
GITSG 1988[50] (24/24)	***SMF*** SMF	54 -	6.5 5.1	p < 0.02
Klaassen[52] (44/47)	***5-FU*** 5-FU	40 -	8.3 8.2	n.a.
Chauffert[53] (59/60)	***5-FU ***(PVI)***/Cis***+Gem Gem	60 -	8.0 14.5	p = 0.03
Loehrer[55] (36/38)	***Gem ***+ Gem Gem	50.4 -	11.0 9.2	p = 0.034
Crane[66] (53/61)	***Gem*** ***5-FU ***(PVI)	30 (10#)	11 9	p = n.s.
Li[78] (18/16)	***Gem*** ***5-FU***	50.4	14.5 6.7	p = 0.027
Ishii[63] (20)	***5-FU ***(PVI)	50.4	10.3	n.a.
McGinn[65] (37)	***Gem***	24-42	11.6	n.a.
Shinchi[47] (16/15)	***5-FU***(PVI) Supportive care	50.4 -	13.2 6.4	n.a.
Brunner[67] (40/42)	***Gem/Cis ***+ Gem ***Gem/Cis***	55.8 55.8	13 8	p < 0.0001
Huguet[24] (72/56)	***5-FU ***(PVI)+ as below FolFuGem, GemOx	55 -	15 11.7	p = 0.0009
Kachnic[64] (23)	***5-FU ***+ Gem	50.4	13	n.a.

*: bold and italics indicate chemotherapy given as concurrent chemoradiotherapy #: radiotherapy fraction number; ^$^: a chemotherapy only trial, but very few patients had some radiotherapy; 5-FU: 5-fluorouracil bolus injection unless marked as 'PVI'; Cis: cisplatin; Gem: gemcitabine; Fol: folinic acid; Iri: irinotecan; MMC: mitomycin C; n.a.: not available; n.s.: not significant; Ox: oxaliplatine; PVI: protracted venous infusion, SMF: streptozotocin, mitomycin, 5-fluorouracil.

Many chemotherapy trials included both patients with locally advanced and metastatic disease. However, only those studies with a subgroup analysis for LAPC allow for an indirect comparison with radiotherapy. Four randomised phase III-studies are suitable for such a comparison [56-59] and the achieved median OS times ranged between 6 and 12 months with a 1-year survival rate of 15%. More recently 3 phase III-studies which randomised between gemcitabine monotherapy and gemcitabine based chemotherapy combinations and which stratified for LAPC and metastatic disease [60-62] have been reported. Median OS was between 8.7 and 11.7 months in the LAPC subgroups. Recent phase II-studies and cohort studies employing CRT reported median OS times of 10-11 months and 1-year-survival rates of up to 40% [47,63-66]. The value and particularly the toxicity of additive chemotherapy before or after CRT have to be further assessed in studies. Combinations of CRT and chemotherapy reported median OS times of 13 - 15 months [24,64,67]. Currently this approach is further evaluated in the international phase III LAP07 trial randomising patients with LAPC who do not progress after four months of chemotherapy with gemcitabine between a CRT with concurrent capecitabine versus two more cycles of gemcitabine.

Resectability should be re-evaluated 6-8 weeks after completion of CRT to exploit the possibility of a curative resection [39,68-73] but it should also be noted that tumour response may appear several months after CRT is completed. Curative resection (R0) can be performed in 10-20% of the patients who initially presented with LAPC.

#### Summary

A direct comparison of CRT and chemotherapy is currently difficult. After the recent presentation of the data from the ECOG 4201 trial this result is pointing to superiority of CRT but requires further validation in clinical trials to test if modern-technique chemoradiotherapy is indeed superior to chemotherapy for LAPC because of the small sample size due to poor accrual. However, as expected toxicity is higher with chemoradiotherapy and should be reduced by optimised techniques. Additive chemotherapy before or after CRT needs to be tested in randomised studies. The most important argument for CRT is a 10-20% rate of secondary resectability. This has not been reported with chemotherapy alone.

## Clinical consequences

-After R0 resections, the current evidence supports the use of adjuvant chemotherapy rather than chemoradiotherapy followed by chemotherapy, even if chemoradiotherapy is regarded as the standard of care in North America.

-After R1-resections adjuvant chemotherapy followed by chemoradiotherapy should be considered.

-Evidence for the role of neoadjuvant chemoradiotherapy is expected from currently open randomised trials and eligible patients should be offered entry into these trials.

-In order to overcome the poor prognosis associated with locally advanced disease it is important to confront the nihilistic beliefs of clinicians. It should be remembered that CRT confers a secondary resectability rate of 10 - 20% offering these patients the prospect of curative treatment.

## Conflict of interests

The authors declare that they have no competing interests.

## Authors' contributions

TBB and MSB: conception, design. All the listed authors have been involved in drafting or in revising the manuscript. All authors read and approved the final manuscript.
